# Malignancy Risk Models for Oral Lesions

**DOI:** 10.4317/medoral.18374

**Published:** 2013-05-31

**Authors:** Ana M. Zarate, María M. Brezzo, Dante G. Secchi, José L. Barra, Mabel Brunotto

**Affiliations:** 1 Research Professor,Departamento de Biología Bucal. Facultad de Odontología, Universidad Nacional de Córdoba-Argentina; 2 Professor, Departamento de Patología Bucal. Facultad de Odontología, Universidad Nacional de Córdoba-Argentina; 3 Research Professor Facultad de Ciencias Químicas - CIQUIBIC-CONICET, Universidad Nacional de Córdoba-Argentina

## Abstract

Objectives: The aim of this work was to assess risk habits, clinical and cellular phenotypes and TP53 DNA changes in oral mucosa samples from patients with Oral Potentially Malignant Disorders (OPMD), in order to create models that enable genotypic and phenotypic patterns to be obtained that determine the risk of lesions becoming malignant. Study Design: Clinical phenotypes, family history of cancer and risk habits were collected in clinical histories. TP53 gene mutation and morphometric-morphological features were studied, and multivariate models were applied. Three groups were estabished: a) oral cancer (OC) group (n=10), b) OPMD group (n=10), and c) control group (n=8). Results: An average of 50% of patients with malignancy were found to have smoking and drinking habits. A high percentage of TP53 mutations were observed in OC (30%) and OPMD (average 20%) lesions (p=0.000). The majority of these mutations were GC ? TA transversion mutations (60%). However, patients with OC presented mutations in all the exons and introns studied. Highest diagnostic accuracy (p=0.0001) was observed when incorporating alcohol and tobacco habits variables with TP53 mutations. Conclusions: Our results prove to be statistically reliable, with parameter estimates that are nearly unbiased even for small sample sizes. Models 2 and 3 were the most accurate for assessing the risk of an OPMD becoming cancerous. However, in a public health context, model 3 is the most recommended because the characteristics considered are easier and less costly to evaluate.

** Key words:**TP53, oral potentially malignant disorders, risk factors, genotype, phenotype.

## Introduction

Non-communicable chronic diseases (NCCD), such as oral cancer, are the leading causes of death in the world (1). Eighty percentages of deaths were caused by NCCD, occurred in low- and middle-income countries, and can be prevented by prevention programs and early diagnosis (2). The challenge of multifactorial phenotypes is to achieve a valid strategy for identifying risk individuals in the population (3).

Oral cancer (OC) is often preceded by lesions with malignant signs, known as oral potentially malignant disorders (OPMD). OPMD generate a wide diversity of symptoms and signs, making it difficult to identify early malignant changes, and the risk factors for OPMD are not the same in different countries and regions (4-6). Predicting the likelihood of malignant transformation of OPMD is thus a major concern for public health stra-tegies, especially in developing countries.

Patients who have been diagnosed at an earlier stage of OPMD require less aggressive treatment, present less morbidity and require lower health costs. The most va-luable tool is the recognition of individual risks, which depends on the availability of meth-odological strategies to identify phenotype-genotype profiles (7,8), hence our interest in studying the particular forms in patients with OPMD or OC. Non-invasive and easily applicable methods such as cytology, associated with other techniques such as well-known TP53 mutations, clinical statements and statistical models could provide data to be used for the generation of risk models (9,10). Even nowadays, there are no reliable marking patterns that describe the risk factors of OPMD becoming OC.

The building of a theoretical model based on basic clinical concepts is essential to evaluate the biodiversity of human health and also re-evaluate prediction methodologies to develop new methods for early presumptive diagnosis. This model should be founded on current conceptions of epidemiology and biostatistics, in order to understand the conditions and mechanisms underlying the development of complex chronic diseases.

So the aim of this work was to assess risk habits, clinical and cellular phenotypes and TP53 DNA changes in oral mucosa samples from patients with OPMD, in order to create models that enable genotypic and phenotypic patterns to be obtained that determine the risk of lesions becoming malignant.

## Material and Methods

-Patients 

A cross-sectional study was conducted of a male-female population (n=28), aged 23-84, attended at the Clinical Office of the Stomatology Clinic “A”, (Faculty of Dentistry, National University of Cordoba, Argentina) between January 2010 and January 2011. This study was approved by the Research and Ethics Committee of the Ministry of Health of the province of Cordoba (No. 1378) and informed consent forms were signed by all patients.

Patients were excluded who were under therapeutic medication such as corticosteroids or chemotherapy drugs that modify or alter the clinical behavior of malignant oral lesions. Patients diagnosed with other cancers, systemic diseases, chronic alcoholism and drug addiction were also excluded.

Three groups were established: a) oral cancer group (OC), b) oral potentially malignant disorders group (OPMD), and c) control group (Con). The OC and OPMD groups were diagnosed by routine histopathological analysis; the Con group diagnosis was performed by other diagnostic methods.

OC group: patients (n=10) with a diagnosis of oral squamous cell carcinoma and?or verrucous carcinoma (ICD-10 C00-C06).

OPMD group: patients (n=10) with a diagnosis of OPMD according to criteria described by Warnakulasuriya et al. (4), who consider OPMD as a family of morphological alterations among which some may have an increased potential for malignant transformation; there are precancerous lesions (leukoplakia, erythroplakia, palatal lesions in reverse smokers) and precancerous conditions (submucous fibrosis, actinic keratosis, lichen planus, discoid lupus erythematosus). A precancerous lesion is morphologically altered tissue in which oral cancer is more likely to occur than in its apparently normal counterpart; a precancerous condition is a generalized state associated with a significantly increased risk of cancer (4).

Con group: patients (n=8) with oral lesions excluding cancer or OPMD.

-Examination of the oral cavity 

The examination was performed by previously calibrated dentists (Kappa = 0.62), through visual inspection and palpation of oral mucosa, teeth and prosthetic devices (removable?fixed). Tongue, lip and cheek parafunction habits were also registered. Patients were asked about lifestyle, age, gender, ethnicity, smoking and alcohol habits and other information. Following Piemonte et al. (11), smokers are those who consumed at least one cigarette per day for a minimum period of one year; drinkers are patients who consume two measures of alcohol per week for a minimum period of one year.

-Exfoliative Cytology

Two smears from the lesion were collected using cytobrush Medibrush ®Plus, Medical Engineering (Argentina). One of the smears was fixed in 96% alcohol for 1 h, and then Papanicolaou stained (12). The other smear was stored in sterile H2O at - 80°C for DNA extraction. For morphometric analysis, 30 cells from each patient were selected. The cells were evaluated using an Olympus BX50 optical microscope and the images were captured with a SONY video camera and Image-ProPlus software, 1999. For the total amount of cells that were selected from each group of lesions (n=300), the nuclear area (NA), cytoplasmic area (CA), and NA/CA ratios were measured using Autocad 2000 software. Only clearly defined cells were measured in each smear and the mean value of NA and CA was obtained for each case. In all cases, the cytoplasmic area and nucleus area were measured in square micrometers.

-DNA isolation and Polymerase Chain Reaction (PCR)

DNA was extracted according to the amended protocol described in Brunotto et al. (13). Cells collected from brushing lesion were resuspended in 2:5 of 70% alcohol and sterile water. They were then centrifuged at 10000 rpm and afterwards incubated in 7?l of proteinase K (1mg/ml) in 150?l of extraction buffer.for 30 min at 50ºC. They were later centrifuged for 5 min at 14000 rpm, the supernatant was recovered and 0.8-1 volume of isopropyl alcohol was added. It was later centrifuged again, eliminating the supernatant, and washed in 70% alcohol for centrifugation for 5 min at 10000 rpm. Finally, the DNA was resuspended in 150 ?l of 1x buffer TE(Tris/EDTA). The 260/280 absorbance ratio of the DNA employed for PCR was ? 1.70. Primers were designed in order to amplify the following regions of the human TP53 gene (GeneBank: NC_000017):

?exons 5-6 Forward: 5’-ACAGTACTCCCCTGCCCTCAACAA-3’, 

Reverse: 5’-CCCAGTTGCAAACCAGACCTCAG-3’

?exons 7-8 Forward: 5’- TGTGTTATCTCCTAGGTTGGCTCTGACT-3’.

Reverse: 5’-TGCTTGCTTACCTCGCTTAGTGCTC-3’ 

The PCR was obtained in a 50 ?l final volume. PCR amplification was carried out on BioRad’s iCycler thermal cycler, using the following protocol: 10 min at 95ºC, 1 min at 95ºC, 1 min at 62ºC, and 2 min at 72ºC for 40 cycles, with an additional 10 min at 72ºC after the last cycle. The PCR products were separated on 0.8% TBE (Tris/Borate/EDTA) agarose gel and stained with ethidium bromide. A DNA ladder marker (Promega, USA) was used to determine the size of DNA fragments. The fragments were purified and sequenced at Macrogen Inc., Seoul, South Korea.

-Statistical Analysis and Models

**Figure 1:**
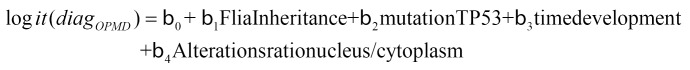
Model 1 (M1).

**Figure 2:**
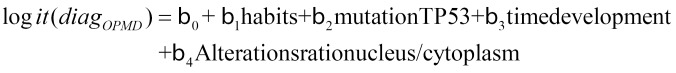
Model 2 (M2).

**Figure 3:**
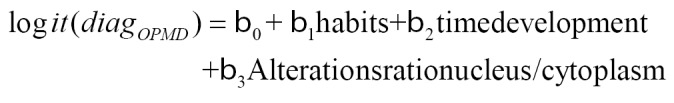
Model 3 (M3).

Data analysis was as follows:

a) The Chi Square Test was performed to evaluate associations or difference of proportions among the groups, setting p<0.05 for statistical significance.

b) The Kappa coefficient was calculated to evaluate the concordance between dentists, setting a value equal to or higher than 0.6 for good concordance.

c) Logit Regression (LR) models were built combining different covariables, in order to discriminate main factors between OC and OPMD. Age, gender, cellular phenotype (presence of cellular atypia in oral smears), genotype (mutations in exon-introns 5 to 8 TP53 DNA sequence), and smoking/alcohol consumption habits in patients were included as predictor variables. The malignant lesions variable was the binary outcome (OPMD: 1; OC: 0). The models built were: (Figs. 1,2,3).

d) The diagnostic accuracy of each model was assessed by the Area Under of the Receiver Operating Characteristic (AUC of ROC curve) estimated by non-parametric methods (14).

The parameters were estimated by Monte Carlo, which was applied because of the small sample size (15).

## Results

-Clinical and biodemographic characteristics 

In the Con group there was a high percentage of female patients (62.5%), while in the OC and OPMD groups most patients were male (p=0.000) ( Table 1). The majority of patients were 45 years of age or more, principally in the OC and OPMD groups (p=0.0007) ( Table 1). Patients with OPMD lesions (n=10) presented oral leukoplakia (OL, 40%) and oral lichen planus (OLP, 60%). The age of the lesion was greater than a year in all groups (p=0.0766) ( Table 1). An average of 85% of patients with OPMD and OC were found with smoking and drinking habits (p=0.000) ( Table 1).

**Table 1:**
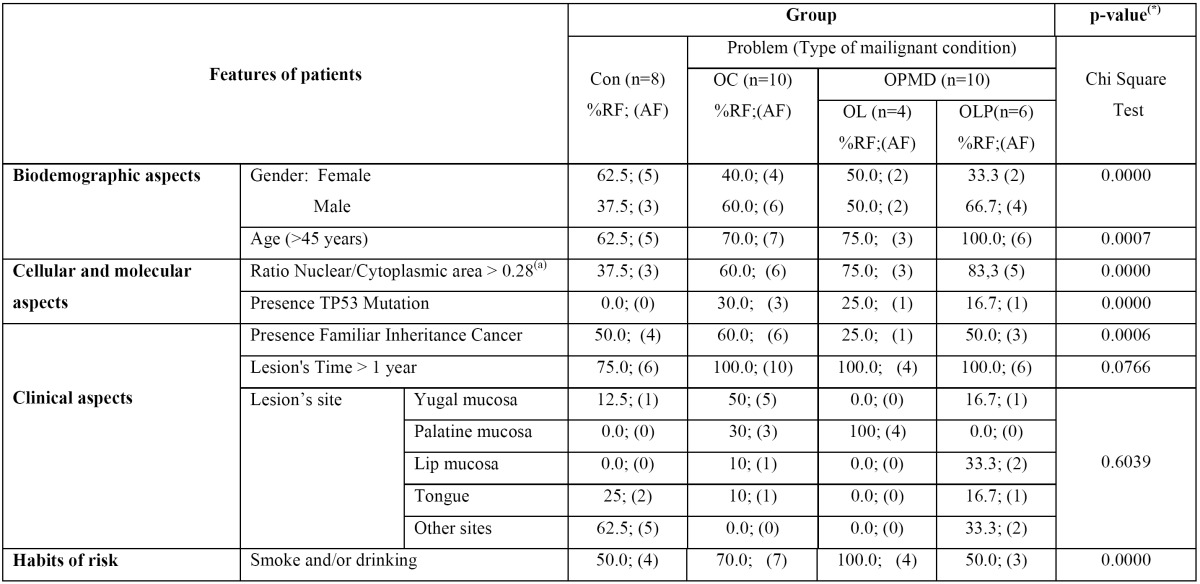
Features of patients under study. OL: Oral Leukoplakia; OC: Oral Carcinoma; OLP: Oral Lichen Planus; OPMD: Oral Potentially Malignant Disorders; Con: control (*) Estimated by Monte Carlo based 10000 sampling tables with initial seed 2000000, setting p<0.05 by statistical significance of association among lesions and features. (a) Median value used as cut-point. All adjusted for age and gender. %RF: % of relative frequencies of patients with features in relation to total of patients in the category; AF: absolute frequencies of patients in the category.

-Cellular and molecular characteristics

Morphometric analysis showed normal features in the NA/CA ratio, and significant proportions of OC patients in the Con and OPMD groups (p=0,000) ( Table 1).

A high percentage of TP53 mutations were observed in OC (30%) and OPMD (average 20%) lesions (p=0.000) (Table 2). Most of these are guanine-cytosine to thymine-adenine (GC ? TA) transversion mutations (60%). In contrast, patients with OC presented mutations in all the exons and introns studied ( Table 2).

**Table 2:**
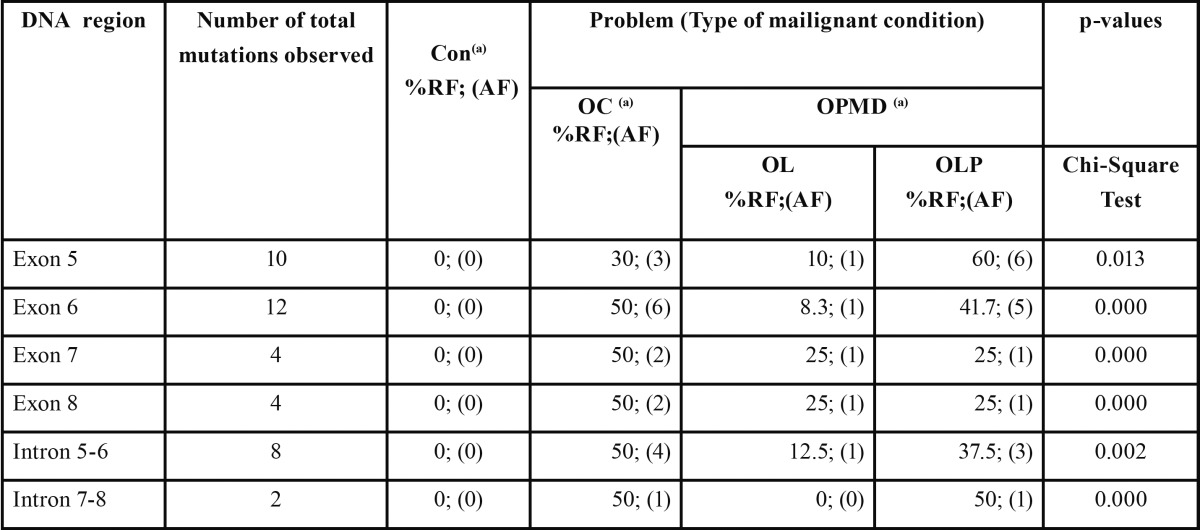
Patients with different kind of lesions presented mutation in the DNA sequenced. Each patient has sequenced the 5-8 exons. Bp: base pair; Con: control; OL: Oral Leukoplakia; OC: Oral Carcinoma; OLP: Oral Lichen Planus; OPMD: Oral Potentially Malignant Disorders. %RF: % of relative frequencies of change bases in relation to total of change bases in the category; AF: absolute frequencies of change bases in the category. (a) Chi-Square Test among proportions of exons/introns, p-values estimated by Monte Carlo, initial seed= 2000000, setting p<0.05 by statistical significance among porportions.

-Prediction models

All estimated AUCs showed a high diagnostic accuracy for each model built. Highest diagnostic accuracy was observed when incorporating the TP53 mutations, NA/CA ratio alterations, and alcohol and tobacco habits variables (98%) ( Table 3).

**Table 3:**
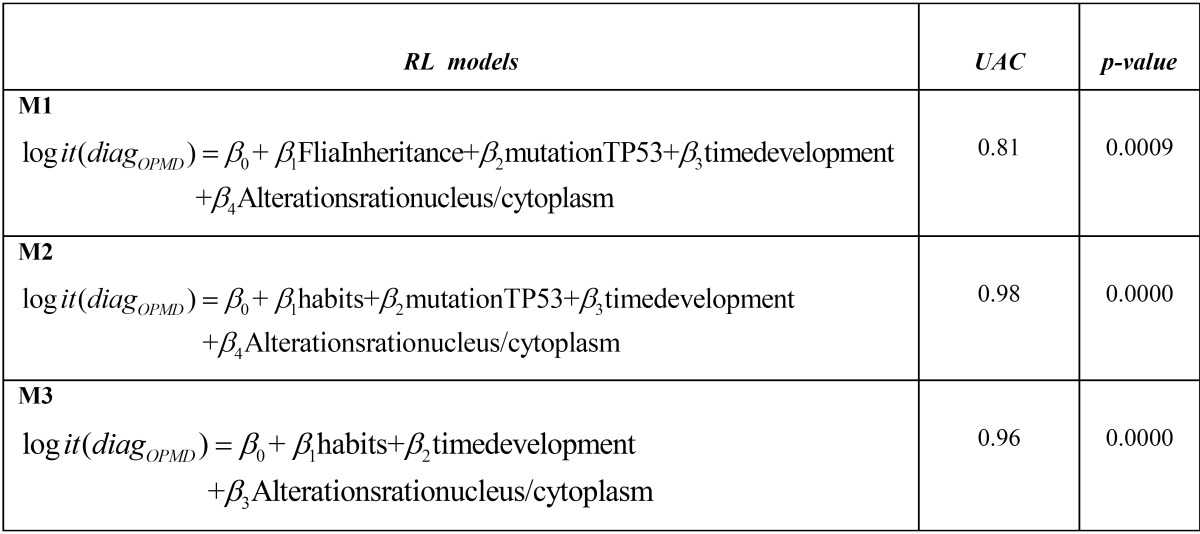
Partial models of Regression Logit (RL) were built, Under Area ROC Curve (UAC) calculated with estimated RL probabilities and the corresponding p-value of UAC (estimated by non parametric methods). All models were ajusted by gender and age. M: model. OPMD: Oral Potentially Malignant Disorders.

## Discussion

Nowadays, there is a focus on complex diseases, with researchers seeking to improve the accuracy of early diagnosis of these pathologies, since dissimilar phenotypes can be obtained in similar cellular and symptom conditions. For that reason, this study combined different variables, such as clinical phenotypes and genotypes as well as habits, in order to create models that enable genotypic and phenotypic patterns to be obtained in patients with OPMD, using non-invasive methods such as exfoliative cytology (16). We included oral lichen planus lesions as OPDM. This is controversial because the major problem of interpretation of malignant potential studies of this disease is the inexistence of strict diagnostic criteria for differentiating lichenoid processes (17). Even though the WHO classifies OLP as a premalignant condition, the underlying mechanisms initiating the development of cancer in OLP lesions are still not understood (18).

It is known that surgical biopsy is the definitive method for diagnosing oral lesions, but this is invasive and has technical constraints and psychological implications for patients. It is impractical to perform a biopsy routinely within screening programs or follow-up control studies (19). Our results obtained through the morphometric study showed that the proportion of OC patients with a nucleus/cytoplasm ratio greater than 28 micras is higher than in other groups studied. Diniz-Freitas et al. (20), who studied oral smears from healthy patients and patients with oral carcinomas, concluded that neither the cytoplasm area, the nucleus area, or the ratio between them are significant parameters to differentiate between oral smears of patients. In addition, Ramaesh et al. (21), using nuclear and cytoplasmic diameter, found no difference between normal mucosa and dysplastic lesions of the oral mucosa. However, Mehrotra et al. (22) consider that this method is important for detecting these lesions and predicting their progression or recurrence. On the other hand, Navone et al. (23) demonstrated that exfoliative cytology presented a sensitivity of 86.5% and an accuracy of 89.6% in detecting patients with dysplasia and/or carcinoma. They found that the single case that was histologically negative at the onset proved positive at cytology. All this strongly suggests that cytomorphometric features are not relevant by themselves as a test of prevention or diagnosis, but this methodology is useful for collecting cells for several analyses and, accompanied by other clinical and molecular signs, should be used as a non-invasive strategy to uncover the nature of cancer if a lesion is clinically benign or potentially malignant (24).

We observed TP53 mutations on exons 5-8 in OPMD (10%) and OC (15%). Ogmundsdóttir et al. (25) confirm these results, since they observed that TP53 mutations can exist in benign oral mucosal lesions or in the recurrence of oral squamous cell carcinoma lesions. In follow-up studies of patients with oral, larynx, hypopharynx and oral squamous cell carcinomas, most patients who had a family history of cancer presented mutations in the TP53 gene in germline cells (26). The presence of mutations in the specific DNA binding site is one compulsory indicator characteristic of a patient deemed to be at risk that should be incorporated into a monitoring program. Moreover, the TP53 marker used in this work is well-known and its usage widespread, involving a lower economic cost than others, and it does not need specialized staff to interpret its results.

In our study, we observed that a significantly high percentage of patients with OC and OL had smoking and drinking habits. These results match those of other investigations in which smoking and drinking habits have been strongly linked to the presence of OC and OPMD (11).

Considering the multifactorial concept of OC and OMPD (5,11), we built logistic models from genotype and phenotype variables that allow patients with OPMD and OC to be classified, using statistical tools to determine the probability of including a patient in either group. When considering variables such as tobacco and alcohol habits and the presence of the TP53 mutation in exons 5-8, the accuracy of the probability of classifying patients increased to 94-96%. It is known that tumorigenesis involves several steps, the first one being the sequential accumulation of genetic mutations, closely related to environmental determinants, followed by a clonal proliferation (5).

Prevention of cancer offers the greatest public health potential and the most cost-effective long-term method of cancer control; besides, early detection helps reveal the presence of the disease at an initial stage, when it has a high potential for cure. The assessment of the value of diagnostic indicators, such as symptoms and genetic testing, enables the sensitivity and specificity of these indicators for determining the presence/absence of a malignant pathology to be estimated. Lingen et al. (27) considered that a description of biomarker profiles is necessary and determine a correlation of changes in biomarker profiles over time in relation to the progression from OPMD to cancer.

We show that patients with OPMD share features with OC patients such as clinical patterns, TP53 changes and drinking and tobacco habits, and thus it would be interesting to conduct multivariate studies to analyze a set of variables.

We also propose a model of malignancy in OPMD, indicating the main features of indicators of oral cancer risk. This model is supported by the genetic alterations that have been shown to contribute directly to malignant development and progression and which are central to understanding the pathway of genes as well as the various phenotypes they generate (27). Both epidemiological (28) and experimental studies (29) indicate that tobacco and alcohol consumption is a risk factor for developing OC. Supporting these findings, research performed in experimental models showed that cigarette smoke exposure leads to quantitative increases in DNA-binding activities of p53 and other proteins (29).

The statistical methods show that the results are reliable because they produce parameter estimates that are nearly unbiased even for small sample sizes. In our previous research conducted from 2000 to 2007 in a population of 406 subjects, 16% (65 patients) presented oral cancer and 11% (45 patients) were diagnosed with OPMD. These data represent about 6 to 10 patients per year with OPMD or OC respectively (11).

Our results permit us to conclude that the presence of TP53 mutation in exon-introns 5 to 8, associated with the presence of habits of alcohol and smoking, may be considered as main risk factors of malignancy transformation.

Models 2 and 3 were the most accurate for assessing the risk of OPMD becoming cancer. However, in the public health context, model 3 is most recommended because the characteristics considered are easier and less costly to evaluate.
